# Around the Hospitals

**Published:** 1894-07-21

**Authors:** 


### Around the Hospitals.

[Notice to the Managers of Institutions.—Special space is now reserved for the insertion of notices of hospital meetings and festivals. The Editor requests that all notices may reach The Hospital Office, 428, Strand, at least one week before the date of each meeting.]

**The Southport Infirmary.**—This is not a large institution, but some excellent work is done nevertheless, and the various entertainments and collections held on its behalf testify to the appreciation in which it is held in the neighbourhood. During the past year an increase of 62 in-patients is shown, and if this increase continues, the new buildings will be completed none too soon. The funds are not yet subscribed to complete the work, and besides this £3,000 will be required for furnishing. It is proposed to hold a bazaar in November towards this object. During the past year 458 in-patients were treated, out-patients numbering 2,134.

**The National Hospital for the Paralysed and Epileptic.**—The work of this hospital is one of peculiar interest, as it affords relief and often cure to sufferers from diseases of a peculiarly trying nature, and further, connected with the hospital is the pension scheme for hopelessly incurable cases of paralysis and epilepsy. The hospital itself does not limit the time of treatment, if good can be effected by prolonged residence or attendance. In the out-patients' department, for example, there is a patient attending who has been a visitor for 30 years, whilst an in-patient is non-convalescent who was an inmate during a trying illness of two years. The aim of the hospital is to cure or relieve as may be possible is thus thoroughly carried out, a condition most necessary in the treatment of the diseases admitted to its walls. The monotonous lives of the patients are cheered in winter through the kindness of those who hold entertainments for them, and many are sent to seaside homes when convalescent, or assisted on leaving by the Samaritan branch of the institution. During the year 1893 the largest number of patients yet admitted were treated, amounting to 966, the out-patients numbering 4,627, also showing an increase. Where it is possible, patients are required to contribute towards their own support, and a guinea weekly is charged in the wards of the new hospital, which secure a certain amount of privacy, whilst separate rooms can be provided for at a higher rate of payment. The contributions by paying patients amounted to £1,988 12s. 6d. last year. The hospital has many generous friends, and was fortunate during the past year in receiving £1,000 donation at the festival dinner from the Earl of Dudley, in commemoration of the escape of his brother, a midshipman on the "Victoria." His lordship gave £60 in addition to the hospital. The hospital is in need of further annual subscriptions. That the ordinary expenditure cannot depend on legacies is shown by the fact that these fell short last year, and caused a deficiency of £3,092 on the accounts, yet both annual subscriptions and donations were larger than in the previous year, and £2,500 was contributed at the festival dinner. The treatment of patients at the National Hospital is, of necessity, an expensive one, yet the cost per occupied bed did not exceed £69 15s. 3d. The gross receipts amounted to £12,737, and the expenditure to £14,030.

